# Breaking Down Listening Barriers in Education: From Hearing Access to Inclusive Communication

**DOI:** 10.3390/audiolres16050148

**Published:** 2026-09-30

**Authors:** Giuseppe Chiarella, Sandro Burdo, Giacinto Asprella Libonati

**Affiliations:** 1 Unit of Audiology, Phoniatrics and Vestibology, Regional Centre for Cochlear Implants and ENT Diseases, R. Dulbecco University Hospital, Department of Experimental and Clinical Medicine, Magna Graecia University, 88100 Catanzaro, Italy; 2 Associazione Italiana Liberi di Sentire, 21100 Varese, Italy; sanburdo@me.com; 3 ENT Unit, Santa Maria delle Grazie Hospital, ASM Matera, 75100 Matera, Italy; giacintoasprella@gmail.com

Education is fundamentally dependent on access to communication. In classrooms and lecture halls, however, access to spoken information cannot be assumed to be equally available to all students. Background noise, reverberation, distance from the speaker, competing voices, unfavorable seating positions, and limited visual access to the speaker can substantially degrade the communication signal. These challenges may be particularly pronounced for students who are deaf or hard of hearing (DHH), but they are not confined to individuals with clinically diagnosed hearing loss. Even students with normal audiometric thresholds may experience difficulty understanding speech under acoustically demanding conditions, especially when sustained listening is required.

This distinction between hearing sensitivity and functional listening ability is central to the present Special Issue, entitled “Breaking Down Listening Barriers for Students with Hearing Difficulties”. Contemporary evidence increasingly indicates that conventional audiological measures alone cannot fully capture the challenges encountered in complex educational environments. Poor classroom acoustics have been associated with adverse effects on listening, learning, and well-being across educational levels [1], while sustained listening under adverse signal-to-noise conditions may increase listening effort and fatigue [2]. Educational accessibility should therefore be understood not simply as the correction of an auditory deficit, but as the creation of environments in which communication remains accessible despite variability in hearing, listening abilities, linguistic background, communication preferences, and educational context.

The eight contributions collected in this Special Issue approach this challenge from complementary perspectives. Together, they move from the characterization of listening difficulties in real educational settings to spatial hearing, audiovisual speech perception, classroom acoustics, assistive listening technologies, speech-to-text captioning, teacher vocal demands, and the integration of multiple visual accessibility solutions. What emerges is a broader conceptual shift: listening barriers are rarely attributable to a single individual factor. Rather, they arise from the interaction between the listener, the communication environment, the speaker, and the technologies or strategies available to support access.

A particularly important contribution to this perspective comes from Gambacorta et al. [3], who explored hearing and listening experiences among high-school and university students and teachers. Their findings highlight a discrepancy between formally recognized hearing impairment and the broader experience of listening difficulty. Approximately 8–9% of students reported diagnosed or perceived hearing difficulties, while environmental variables also emerged as meaningful barriers: classroom noise was particularly relevant among high-school students, whereas seating position appeared especially important at university level. These observations reinforce an essential point: difficulties in accessing spoken information may remain underestimated when assessment is restricted to conventional measures of peripheral hearing.

This has practical implications for both clinical audiology and education. Functional listening should increasingly be evaluated in contexts that approximate the complexity of everyday communication. Recent efforts to develop classroom-specific patient-reported measures, including instruments designed for children with unilateral hearing loss, similarly reflect the need to capture difficulties arising in different classroom configurations and listening situations [4]. Such approaches complement rather than replace conventional audiological assessment, allowing the consequences of hearing difficulties to be considered from the student’s perspective.

The importance of ecological listening is further illustrated by Aruffo et al. [5], who examined sound localization in young individuals with single-sided deafness (SSD) using a virtual-reality paradigm. Participants with SSD demonstrated substantially greater localization errors than normal-hearing peers and increased head movements suggestive of compensatory strategies. Spatial hearing is particularly relevant to education because classrooms are inherently dynamic auditory environments. Teachers may move while speaking, classmates contribute from different positions, and relevant information often originates outside the student’s frontal visual field. Difficulties in identifying the location of a speaker may therefore influence not only auditory performance but also orientation, attention, social interaction, and participation.

At the same time, communication in educational settings is not exclusively auditory. Human speech perception is intrinsically audiovisual, and the visual components of speech become increasingly important when the acoustic signal is degraded. Battista et al. [6] examine lip-reading within this broader framework, emphasizing its role throughout development and in both typical and atypical communication. Lip-reading should not be regarded simply as a compensatory strategy reserved for individuals with severe hearing impairment. Rather, visual speech information interacts with auditory information as part of normal speech perception and may become particularly valuable in noisy environments.

This perspective has immediate practical consequences. Visibility of the speaker’s face, appropriate lighting, classroom positioning, and communication behaviors that preserve access to facial and articulatory cues should be considered integral components of accessible communication. Conversely, any environmental or technological intervention that improves one communication channel while inadvertently limiting another should be evaluated carefully. Accessible classroom design is therefore not solely an acoustic problem; it is inherently multimodal.

This multidimensional view is particularly evident in the A.BA.CO. project described by Orzan et al. [7]. The project provides a framework for addressing communication barriers through the integration of audiological, acoustic, technological, organizational, and educational perspectives. This systems-based approach represents one of the major themes of the Special Issue. Classroom accessibility cannot be achieved through a single device or isolated accommodation. Acoustic conditions, awareness of hearing and listening difficulties, teaching practices, seating organization, visual accessibility, assistive technologies, and the characteristics and preferences of individual students must be considered together.

Technology constitutes an increasingly important part of this communication ecosystem. Fastelli et al. [8] examined speech-to-text captioning in schools using SWOT and TOWS analyses, identifying both its substantial potential and the barriers that may limit effective implementation. Automatic captioning can expand access to spoken content and may be particularly valuable when the auditory signal is degraded. Nevertheless, technological availability does not necessarily translate into educational effectiveness. Recognition accuracy, latency, personalization, classroom infrastructure, usability, privacy, teacher acceptance, stigma, cognitive load, and integration into routine teaching all influence whether a potentially useful technology becomes genuinely accessible.

These implementation considerations are consistent with experiences reported directly by DHH students using real-time captioning in higher education, where captioning can facilitate communication access but where quality, reliability, and the way services are delivered remain critical determinants of the user experience [9]. Accessibility technologies should therefore be evaluated not merely by whether they function technically, but by whether they improve meaningful participation in the educational setting.

The same principle applies to remote microphone systems. Such systems are designed to improve the effective signal-to-noise ratio by transmitting a speaker’s voice directly to a listener’s hearing technology and have demonstrated benefits for children with hearing loss or auditory processing difficulties [10]. However, Andreatta et al. [11] show that the technical implementation of these systems deserves careful attention. Their analysis demonstrated differences in latency across remote microphones and identified potential frequency–response interactions when hearing-aid and remote-microphone signals are combined. These findings are important because seemingly small electroacoustic differences may influence the perceptual experience of the user. As educational accessibility becomes increasingly technology-mediated, technical validation and user-centered evaluation must therefore proceed in parallel.

Importantly, classroom acoustics affect not only those receiving spoken information but also those producing it. Frangipane et al. [12] explored the relationship between environmental noise and teachers’ voice-related quality of life. Although their investigation was exploratory and involved a limited number of teachers, increasing classroom noise was significantly associated with greater self-perceived vocal difficulty. This observation broadens the concept of acoustic accessibility beyond the student alone.

In a noisy classroom, students may experience reduced speech intelligibility and increased listening effort, while teachers may respond by increasing vocal effort. This interaction can create an unfavorable communication cycle in which greater vocal demand does not necessarily restore an adequate signal-to-noise ratio and may simultaneously increase the speaker’s risk of vocal fatigue. Improving classroom acoustics should consequently be considered both an accessibility intervention for learners and an intervention supporting the occupational well-being and communicative effectiveness of educators.

The final contribution to the Special Issue broadens the discussion from auditory access to the coordination of multiple visual communication channels. Pavani and Leonetti [13] reviewed the evidence concerning the simultaneous use of captions and sign-language interpreting for DHH students. Notably, only four experimental studies met their eligibility criteria, leaving insufficient evidence to determine whether combining these two accessibility solutions consistently improves or impairs learning compared with either solution alone.

This finding is particularly instructive. Accessibility should not be equated with simply increasing the amount of information presented to a student. Multiple concurrent visual streams—including the teacher, slides, captions, and a sign-language interpreter—may compete for visual attention. Their effectiveness is consequently likely to depend on several factors, including individual language preferences, hearing status, age, educational experience, task demands, spatial arrangement of information, and cognitive load. The central question is therefore not whether more accessibility resources should always be provided, but how they can be combined in a way that maximizes access without creating additional perceptual or attentional barriers.

Taken together, the papers in this Special Issue support four closely related principles.

First, **hearing loss and listening difficulty should not be treated as interchangeable concepts**. Audiometric thresholds remain fundamental for identifying peripheral hearing impairment, but they do not fully predict an individual’s ability to follow communication in noisy, reverberant, spatially complex, or cognitively demanding situations. Crucially, traditional audiometry measures hearing sensitivity monaurally—one ear at a time. Consequently, it fails to evaluate how the two ears integrate environmental sounds as they travel through the auditory pathway. Before reaching the cortex for high-level decoding, raw acoustic impulses from each cochlea are processed by the auditory brainstem. Through rapid synapses, cross-lateral pathways, and balanced excitation–inhibition mechanisms, the brainstem utilizes interaural timing and intensity differences to build precise spatial and temporal sound maps. This subcortical binaural integration is what enables listeners to pinpoint sound sources, segregate speech from background noise, and route salient acoustic signals to higher cortical regions. Weaknesses in binaural processing are highly prevalent among typically developing children and are even more pronounced in those with listening and learning difficulties.

Second, **the environment is an active determinant of communication performance**. Noise, reverberation, distance, spatial configuration, visibility, and the characteristics of the communication signal influence whether information is accessible. Barriers therefore cannot always be attributed primarily to the listener.

Third, **educational communication is multimodal**. Auditory information, facial speech cues, captions, sign language, and assistive listening technologies should not be viewed as competing philosophies of accessibility. They represent different—and potentially complementary—channels whose relative value depends on the needs and preferences of the individual learner and on the task.

Fourth, **implementation is as important as technological capability**. A technically sophisticated intervention can provide limited benefit if it is unreliable, poorly integrated into classroom practice, cognitively demanding, stigmatizing, or inconsistent with the user’s communication preferences. Conversely, relatively simple environmental or communication modifications can have substantial effects when they address the barriers that students actually encounter.

These principles encourage a transition from an accommodation-based model, in which solutions are added after a difficulty has been identified, toward inclusive communication design, in which learning environments are developed from the outset to provide multiple effective routes to information.

Several priorities emerge for future research.

A first priority is greater ecological validity. Speech recognition measured under controlled laboratory conditions remains essential, but future studies should increasingly assess outcomes reflecting everyday educational experience, including listening effort, fatigue, comprehension, sustained attention, classroom participation, social interaction, well-being, and academic performance. However, the evaluation and interpretation of these complex, higher-level outcomes will remain fundamentally limited unless subcortical processes—specifically binaural integration—are systematically accounted for, measured (e.g., through dichotic listening tasks), and addressed prior to investigating advanced speech and communication metrics.

A second priority is longitudinal research. Most available evidence characterizes communication performance or technological benefit at a particular point in time. Less is known about how persistent listening difficulty influences educational trajectories, fatigue, participation, psychosocial well-being, or the long-term use and acceptance of assistive technologies. Longitudinal studies will be essential to determine whether improvements in access translate into durable educational and social benefits.

Third, future research should move toward personalized accessibility. Students with similar audiograms may differ substantially in spatial hearing, speech-in-noise performance, listening effort, language experience, visual communication preferences, technology use, and educational needs. No single accessibility solution can therefore be assumed to be optimal for all DHH students or all students reporting listening difficulties.

Fourth, accessibility research should include greater co-design with end users. Students, families, teachers, audiologists, speech and language professionals, educators, engineers, interpreters, and accessibility specialists should be involved not only in evaluating interventions but also in defining the problems that those interventions are intended to solve. User-centered design is particularly important for technologies whose effectiveness depends heavily on acceptability and consistent use.

Finally, rapidly evolving artificial intelligence (AI) and automatic speech-recognition technologies are likely to transform educational accessibility. AI-based captioning, speech enhancement, adaptive interfaces, and personalized assistive tools already show potential to improve communication and educational inclusion for DHH learners [14]. However, technological development should not outpace evaluation. Accuracy across speakers and languages, latency, privacy, bias, reliability, cognitive load, accessibility, and the risk of replacing rather than supporting human communication must remain central considerations.

**Conclusions**

The collective message of this Special Issue is that access to education depends on access to communication, which is determined by considerably more than hearing thresholds alone.

The contributions gathered here demonstrate how listening barriers may arise from the interaction among individual auditory abilities, spatial hearing, classroom acoustics, visual speech information, teaching conditions, technological characteristics, and the organization of accessibility resources. They also show that effective solutions are unlikely to emerge from any single discipline.

The next step is therefore not simply to develop more technologies or more accommodations, but to determine which combination of environmental, auditory, visual, technological, and educational strategies works best, for whom, and under which conditions. Achieving this goal will require ecologically valid assessment, interdisciplinary collaboration, meaningful involvement of students and educators, and a commitment to evaluating accessibility through real-world educational outcomes.

Breaking down listening barriers ultimately means moving beyond the idea that students must adapt to the communication environment. The longer-term objective should be to create educational environments that are themselves designed to adapt to the diversity of those who learn within them.
